# Right Ventricular Dynamics in Tricuspid Regurgitation: Insights into Reverse Remodeling and Outcome Prediction Post Transcatheter Valve Intervention

**DOI:** 10.3390/ijms26136322

**Published:** 2025-06-30

**Authors:** Philipp M. Doldi, Manuela Thienel, Kevin Willy

**Affiliations:** 1 Medizinische Klinik und Poliklinik I, LMU Klinikum, Marchioninistraße 15, 81377 Munich, Germany; manuela.thienel@med.uni-muenchen.de; 2 Kliniken für Kardiologie II & III, Universitätsklinikum Münster, 48149 Muenster, Germany; kevin.willy@ukmuenster.de

**Keywords:** right ventricular remodeling, transcatheter tricuspid valve intervention, right ventricular dynamics

## Abstract

Tricuspid regurgitation (TR) represents a significant, often silently progressing, valvular heart disease with historically suboptimal management due to perceived high surgical risks. Transcatheter tricuspid valve interventions (TTVI) offer a promising, less invasive therapeutic avenue. Central to the success of TTVI is Right Ventricular Reverse Remodelling (RVRR), defined as an improvement in RV structure and function, which strongly correlates with enhanced patient survival. The right ventricle (RV) undergoes complex multi-scale biomechanical maladaptations, progressing from adaptive concentric to maladaptive eccentric hypertrophy, coupled with increased stiffness and fibrosis. Molecular drivers of this pathology include early failure of antioxidant defenses, metabolic shifts towards glycolysis, and dysregulation of microRNAs. Accurate RV function assessment necessitates advanced imaging modalities like 3D echocardiography, Cardiac Magnetic Resonance Imaging (CMR), and Computed Tomography (CT), along with strain analysis. Following TTVI, RVRR typically manifests as a biphasic reduction in RV volume overload, improved myocardial strain, and enhanced RV-pulmonary arterial coupling. Emerging molecular biomarkers alongside advanced imaging-derived biomechanical markers like CT-based 3D-TAPSE and RV longitudinal strain, are proving valuable. Artificial intelligence (AI) and machine learning (ML) are transforming prognostication by integrating diverse clinical, laboratory, and multi-modal imaging data, enabling unprecedented precision in risk stratification and optimizing TTVI strategies.

## 1. Introduction

Tricuspid regurgitation (TR), particularly its functional form, represents a significant and increasingly recognized valvular heart disease. This condition, prevalent in approximately 25% of individuals aged 75 and older with moderate to severe TR, often progresses silently, with patients remaining asymptomatic until advanced stages [1]. Functional TR accounts for nearly 90% of all TR cases and primarily stems from the dilation of the right ventricle (RV) or right atrium (RA) or both, frequently secondary to left-sided heart disease, atrial fibrillation, or pulmonary hypertension. This pathological remodeling leads to tricuspid annular dilation and leaflet tethering, exacerbating regurgitation [2].

The integrity of RV function is a critical determinant of morbidity and mortality in patients afflicted with TR and associated conditions such as pulmonary hypertension [2]. Historically, the RV has been termed the “forgotten ventricle,” receiving less scientific scrutiny than its left counterpart, partly due to its complex and irregular three-dimensional anatomy, which poses considerable challenges for comprehensive assessment [2].

Despite the grave prognosis associated with significant TR, its management has long been suboptimal. Over 90% of patients with severe TR have traditionally not undergone surgical intervention, largely due to the perceived high surgical risks and an outdated belief that all forms of TR would spontaneously resolve following treatment of primary left-sided heart conditions. However, this paradigm is shifting with the advent of transcatheter tricuspid valve interventions (TTVI). These less invasive alternatives, encompassing both repair (e.g., edge-to-edge repair, annuloplasty) and replacement procedures, offer a promising therapeutic avenue for high-risk or inoperable patients [3]. The primary objectives of TTVI are to reduce TR severity, alleviate symptoms, and, crucially, potentially induce right heart remodeling, leading to preserving or improving right heart function.

**Figure 1:**
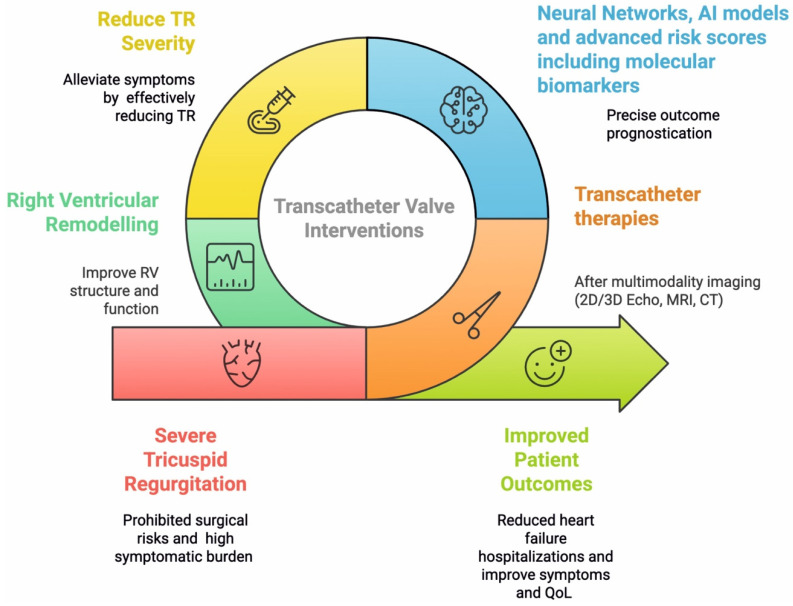
Transcatheter Interventions Induce Right Heart Remodeling.

Central to the success of these interventions is the concept of right ventricular reverse remodeling (RVRR). Defined as an improvement in RV structure and function, RVRR is a critical outcome post-TR reduction, strongly correlated with improved patient survival and a reduced incidence of heart failure hospitalizations (Figure 1) [4]. A thorough understanding of the underlying mechanisms and the ability to accurately predict the occurrence of RVRR are paramount for optimizing patient selection and tailoring therapeutic strategies.

This review aims to provide a comprehensive overview of the pathophysiological mechanisms driving RV remodeling in TR, with a specific emphasis on the biomechanical aspects. It then summarizes the current evidence regarding RV function assessment and the observed patterns of RVRR following transcatheter tricuspid valve interventions. Finally, the critical importance of predicting patient outcomes is discussed, highlighting the emerging and transformative role of neural networks and artificial intelligence (AI) in leveraging biomechanical markers for precise prognostication.

## 2. Pathophysiological Landscape of Right Ventricular Remodeling in Tricuspid Regurgitation

### 2.1. Biomechanical Adaptations and Maladaptive Changes

The right ventricle exhibits remarkable multi-scale biomechanical adaptations in response to pressure overload, such as that seen in pulmonary hypertension, ranging from organ-level changes to alterations at the cellular and fiber levels [5]. Initially, the RV responds by undergoing concentric hypertrophy, characterized by an increase in wall thickness. This adaptive response effectively reduces RV wall stress and maintains organ-level contractility, thereby preserving cardiac output and ejection fraction in the early stages of increased afterload [6]. However, as the underlying pathology progresses and pulmonary artery pressures continue to rise, this compensatory mechanism becomes insufficient. The RV then transitions to eccentric hypertrophy, manifesting as chamber dilation. While this dilation is an attempt to maintain stroke volume and cardiac output, it paradoxically increases wall stress and RV oxygen consumption, ultimately leading to depressed organ-level contractility, reduced cardiac output, and overt RV dysfunction [6].

At the tissue level, pulmonary hypertension induces increased biaxial stiffness in the RV free wall (RVFW), accompanied by a pronounced increase in tissue anisotropy, particularly in the longitudinal direction. This stiffening is further compounded by RVFW fibrosis, characterized by an increased collagen content [5]. Fibrosis can result from capillary rarefaction, reduced RV perfusion, and heightened oxidative stress, leading to a mismatch between RVFW oxygen supply and demand. Increased RVFW collagen content is linked to diastolic dysfunction and reduced RV compliance. Collagen metabolism, which is the balance of collagen synthesis and degradation, is regulated by mechanical loads, and its accumulation (fibrosis) is a common feature of RV failure, though its specific role in different etiologies and stages of heart failure is complex [7,8].

At the cellular level, the initial response to pulmonary hypertension involves a significant increase (30–50%) in the maximum Ca^2+^-activated tension of RV myofilaments [9]. However, with disease progression and the onset of end-stage RV remodeling, this tension reduces, associated with decreased Ca^2+^ sensitivity and alterations in the phosphorylation patterns of contractile proteins [10]. A phenotypic transition from α to β isoforms of myosin heavy chains is also observed, signifying maladaptive remodeling and depressed myocyte contractile dynamics. Cardiomyocyte hypertrophy and an increase in cross-sectional area are noted, particularly in early adaptive stages, with myocyte width increasing in late stages as a morphological marker of RV dysfunction [10]. Additionally, the passive stiffness of myofibers progressively increases, with a notable two-fold rise occurring during early RV remodeling.

The impact of architectural remodeling on RV function and RV-PA coupling is profound (Figure 1). Studies demonstrate that reductions in the helical range of myofibers in the RV free wall are significantly associated with the preservation of circumferential strain and less reduction in RV-PA coupling [11]. This observation underscores architectural changes as a principal driver of RV adaptation to pressure overload [12]. Conversely, maladaptive changes in myo-architecture can impair cardiac motion, leading to RV-PA uncoupling [12]. The interplay between mechanical stimuli and multi-scale remodeling suggests that interventions targeting biomechanical properties, such as reducing wall stress or restoring fiber orientation, could be as crucial as or even more effective than those solely addressing the primary pressure overload. This perspective shifts the focus from merely treating the cause (e.g., pulmonary hypertension) to directly modifying the heart’s structural and mechanical response. Such an approach could open new therapeutic avenues and refine patient selection for TTVI based on specific biomechanical profiles. The identification of a biomechanical metric for the “point of no return” in pulmonary arterial hypertension progression remains a critical area of research.

### 2.2. Molecular Mechanisms of RV Maladaptive Remodeling

It is crucial to differentiate between atrial and ventricular functional TR, as they arise from distinct pathophysiological mechanisms. Ventricular functional TR typically results from left ventricular or pulmonary hypertension, leading to RV remodeling and subsequent tricuspid annular dilation. In contrast, atrial functional TR primarily stems from atrial pathologies, most commonly atrial fibrillation. The resulting chronic atrial dilation leads to right atrial enlargement and subsequent tricuspid annular dilation, even in the absence of significant ventricular disease. The molecular mechanisms underlying these two types of functional TR may exhibit both overlaps and divergences. While both ultimately involve pathways leading to fibrosis and structural remodeling, the specific triggers and the relative importance of different molecular pathways could vary. For instance, inflammatory pathways or specific gene expression changes might be more prominent in atrial remodeling driven by chronic atrial stretch, compared to ventricular pressure overload. A deeper understanding of these distinctions is crucial for tailoring therapeutic strategies [13].

The progression of right ventricular dysfunction from an adaptive state to overt failure in the context of tricuspid regurgitation is driven by a complex interplay of molecular mechanisms. At the forefront of these pathological processes are increased pressure afterload, myocardial hypoxia, and metabolic dysfunction. These factors, often exacerbated by genetic predisposition, inflammation, pharmacological agents, infections, and sex hormones, initiate and perpetuate a vicious cycle leading to adverse pulmonary vascular and cardiac remodeling [14].

A critical molecular aspect differentiating RV from left ventricular (LV) failure lies in the handling of reactive oxygen species. Under afterload stress, both ventricles exhibit increased reactive oxygen species production. However, the RV’s antioxidant defenses, including enzymes like superoxide dismutase (SOD) and glutathione peroxidase (GPX), fail prematurely and are not activated during the compensated stage, unlike in the LV. This inherent deficiency is associated with increased RV susceptibility to ROS-induced damage at an earlier stage than the LV. Furthermore, the primary sources of reactive oxygen species production differ: in RV failure, both NADPH oxidase and mitochondrial complex II activity increase, suggesting a greater contribution from mitochondrial reactive oxygen species generation, whereas in the LV, NADPH oxidase is the predominant source (Table 1). This differential vulnerability to oxidative stress implies that therapeutic strategies for RV failure may require specific targeting of mitochondrial reactive oxygen species and reinforcement of antioxidant pathways, rather than merely extrapolating approaches from LV failure models. This fundamental molecular distinction contributes significantly to the RV’s unique and often rapid pathophysiological trajectory towards failure [15].

Mitochondrial dysfunction and subsequent metabolic reprogramming are also central to RV maladaptive remodeling. RV failure is characterized by a reduction in intact mitochondria, increased variability in their size, structural disarray, and an elevated number of autophagosomes, all indicative of dysregulation in mitochondrial morphology and function. This dysfunction is commonly observed in conjunction with the decreased expression of crucial metabolic master regulators such as PPARγ, PGC1α, and PPARα (Table 1). Under stress, the RV shifts its primary energy substrate utilization from fatty acid oxidation (which normally accounts for approximately 70% of ATP production) towards an increased reliance on glucose through the glycolytic pathway, a phenomenon known as the “Warburg effect”. This metabolic shift occurs despite adequate oxygen supply, leading to reduced ATP generation efficiency. Pyruvate dehydrogenase kinase (PDK) plays a pivotal role in this process; its increased expression inhibits glucose oxidation, contributing to decreased RV contractility. Concurrently, dysfunctional fatty acid metabolism results in lipid accumulation and the production of toxic intermediates like ceramide and palmitate, which can induce lipotoxic cardiomyopathy. Furthermore, insulin resistance and increased glutamine utilization (glutaminolysis) are observed, contributing to the overall metabolic derangement [14,15].

Epigenetic changes, particularly the dysregulation of microRNAs (miRNAs), also play a crucial role in RV maladaptation. Specific miRNAs have been implicated in driving RV failure: miR-143/145 are associated with increased vascular tone, miR-34 is associated with enhanced apoptosis, and miR-379/miR-503 are associated with reduced endothelial cell proliferation. The downregulation of miR-126, for instance, is associated with decreased activation of RAF and MAPK, thereby inhibiting the VEGF pathway. These miRNAs are not merely passive indicators but active participants in the maladaptive process, suggesting a dual potential for their application: as novel therapeutic targets (e.g., Targeted delivery of miR-126 has been observed to improve RV vascularity and function in experimental models) and as non-invasive diagnostic or prognostic biomarkers for RV remodeling and failure. This precise molecular dysregulation offers promising avenues for highly targeted interventions. Other molecular modifiers found to be downregulated or impaired in experimental pulmonary hypertension with maladaptive RV remodeling include hypoxia-inducible factor (HIF)-1α, apelin, angiopoietin 1, insulin growth factor 1, and stromal-derived factor 1. Conversely, elevated levels of insulin-like growth factor binding protein 2 (IGFBP-2) at baseline have been identified as an independent predictor for the non-development of RVRR and persistent TR/RV dilation following transcatheter mitral valve repair, suggesting its potential utility in predicting outcomes for tricuspid valve interventions as well. The PI3K/Akt/mTOR pathway, which is inactivated in pathological hypertrophy, is activated in physiological hypertrophy and has been shown to enhance ventricular function and suppress apoptosis, suggesting a potential pathway for promoting RVRR [14,16]. The collective impact of these molecular aberrations culminates in a sustained inflammatory response and fibrotic remodeling. Elevated levels of pro-inflammatory cytokines, such as TNF-α and IL-6, along with increased activity of matrix metalloproteinases (MMPs), contribute to extracellular matrix disarray and collagen deposition, which stiffen the RV myocardium and further impair its compliance and contractility. This complex molecular cross-talk creates a feedback loop where the initial insults trigger maladaptive signaling cascades, perpetuating oxidative stress, metabolic dysfunction, and structural changes, ultimately driving the RV towards decompensation and overt failure.

## 3. Assessing Right Ventricular Function and Evidence of Reverse Remodeling Post Transcatheter Interventions

### 3.1. Advanced Imaging for RV Function Assessment

Accurate assessment of right ventricular function is crucial for diagnosing TR, guiding interventions, and monitoring the effects of therapy. Given the RV’s complex and irregular geometry, advanced imaging modalities are increasingly indispensable.

Echocardiography remains the first-line imaging tool for both RV dysfunction and TR quantification [17]. Conventional echocardiographic parameters include RV fractional area change (RVFAC), RV myocardial performance index (RVMPI), tricuspid annular plane systolic excursion (TAPSE), and tissue Doppler-derived peak systolic velocity [18]. However, these 2D methods are often limited by suboptimal acoustic windows and reliance on geometric assumptions, which may not fully capture the RV’s complex 3D anatomy, especially when the chamber is enlarged [19].

To overcome these limitations, RV strain imaging, particularly speckle tracking echocardiography, offers a more direct, angle-independent, and reproducible assessment of regional myocardial function [19,20]. This technique is significantly more sensitive for the early detection of RV dysfunction than conventional methods, as myocardial deformation impairment can manifest even before a decline in RV ejection fraction (RVEF) is observed [21]. Reduced RV longitudinal strain, a key metric derived from speckle tracking, has been identified as an independent predictor of decreased survival. The utility of 2D parameters alone might be insufficient to predict outcomes, highlighting the need for more nuanced assessments.

Further enhancing echocardiographic assessment, 3D echocardiography is crucial for a detailed and comprehensive characterization of the RV’s complex and irregular anatomy, particularly when the chamber is enlarged. It provides more reliable volumetric measurements and RVEF estimations compared to 2D methods [22,23] (Table 2). The shift towards advanced 3D imaging (3D echo, CMR, CT) and deformation analysis (strain, tagging) is not merely about achieving better quantification; it represents a fundamental advancement in capturing the RV’s intricate biomechanical behavior. This allows for earlier detection of subtle maladaptations and more precise monitoring of RVRR, which is critical for timely intervention and assessing treatment efficacy. This also implies that future predictive models should prioritize these more nuanced biomechanical markers.

Cardiac magnetic resonance imaging (CMR) is widely regarded as the gold standard for volumetric quantification of the RV [24]. Its superior endocardial definition allows for comprehensive and accurate measurement of RV volumes (end-diastolic, end-systolic), mass, and ejection fraction without ionizing radiation. CMR also enables quantitative measurement of TR severity using phase-contrast images, providing precise data on regurgitant volume and fraction. Furthermore, CMR offers unique insights into regional RV performance through techniques like 1-D myocardial tissue tagging, which can identify decreased shortening in specific RV regions, providing a more granular understanding of myocardial mechanics (Table 2).

Cardiac computed tomography (CT) provides detailed anatomical information of the tricuspid valve and RV [24]. Full-cycle CT is particularly adept at capturing the complex anatomy of the RV and the tricuspid annular plane throughout the cardiac cycle [24]. CT-measured 3D-TAPSE, especially posterior iTAPSE and iTAPSE volume, has demonstrated incremental value in predicting cardiovascular outcomes, including hospitalization and mortality, following TTVI, outperforming conventional 2D-TAPSE measurements. This highlights the importance of detailed 3D biomechanical assessment beyond conventional 2D measures.

### 3.2. Mechanisms and Manifestations of RVRR After TTVI

Transcatheter tricuspid valve interventions fundamentally induce biomechanical changes by reducing the severity of tricuspid regurgitation, thereby decreasing the volume overload and wall stress on the right ventricle. Procedures such as transcatheter edge-to-edge repair (T-TEER) achieve this by coapting leaflets with clips, effectively reducing the regurgitant orifice. Annuloplasty devices, another form of TTVI, reduce tricuspid annular diameters. These direct mechanical modifications limit the backward flow of blood into the right atrium, allowing the RV to pump blood more efficiently into the pulmonary circulation. The direct reduction of tricuspid regurgitation is the immediate mechanical effect of TTVI. This highlights that the primary biomechanical change induced by TTVI is the reduction of volume overload on the RV. This reduction in preload and wall stress then initiates the cascade of reverse remodeling at multi-scales (Table 3). The success of the intervention, therefore, is directly tied to the magnitude of TR reduction, which, in turn, drives the extent of RVRR and subsequent clinical benefits [4,25].

The right ventricular reverse remodeling observed following T-TEER typically follows a biphasic pattern [4,26]. The first phase, characterized by early RV volume unloading, manifests as a significant reduction in right ventricular end-diastolic volume (RVEDV) immediately after the procedure. For instance, studies have shown a 9.7% reduction in RVEDV from baseline to discharge, with this reduction peaking at one year [27]. Following transcatheter tricuspid valve replacement (TTVR), a substantial 35% reduction in RVEDV has been reported. This early phase reflects the immediate hemodynamic benefit of reduced regurgitation. The second phase involves later structural remodeling, where a reduction in right ventricular end-systolic volume (RVESV) begins to manifest later, typically around the 6-month follow-up (e.g., a 5.4% reduction from baseline to 6 months), and continues to progress over time.

Regarding RV function, RVEF may initially show a transient decline immediately post-procedure. However, it gradually increases over time, often returning to pre-procedural baseline values by two years. Importantly, the effective RVEF (calculated as RV stroke volume divided by RVEDV) often improves immediately after TTVI. This biphasic pattern suggests that RVRR is not a simple, linear process (Table 3). The initial decline in RVEF might be a transient response to the sudden reduction in volume overload, as the ventricle adjusts its contractility and geometry to the new loading conditions. The later structural remodeling and recovery of RVEF and strain indicate a more fundamental, adaptive re-organization at the tissue and cellular levels (Figure 1). This understanding is critical for clinical follow-up, as an early drop in EF might be misinterpreted as a negative outcome if the biphasic nature is not appreciated. It also emphasizes the importance of long-term monitoring to fully capture the benefits of RVRR [28].

Beyond volumes, studies consistently demonstrate significant reductions in average RV and tricuspid valve dimensions after TTVR, including a 12.3% decrease in RV end-diastolic area and an 11.9% decrease in RV end-systolic area. RV global longitudinal strain (RV GLS) and RV-PA coupling, assessed by the tricuspid annular plane systolic excursion/pulmonary artery systolic pressure (TAPSE/PASP) ratio, are robust tools for evaluating RV dysfunction and serve as important prognostic factors [29]. RVRR is often defined as an improvement of greater than 10% in either RV FW-GLS or RV-PA coupling from baseline. Significant improvement in RV-PA coupling (e.g., TAPSE/PASP ratio improving from 0.36 to 0.42) is a consistent finding post-TTVI [4].

Furthermore, the reduction in RV volume overload achieved by TTVI positively impacts biventricular interaction. This leads to an alleviation of the leftward bowing of the interventricular septum and an improvement in early left ventricular (LV) filling. Consequently, an increase in LV forward stroke volume (e.g., a 30% increase) is observed, contributing to enhanced overall cardiac output and systemic perfusion [30,31].

## 4. Predicting Outcomes and the Role of Artificial Intelligence

### 4.1. Clinical Importance of Outcome Prediction in TTVI

Patients undergoing transcatheter tricuspid valve interventions for severe TR exhibit significant heterogeneity in their clinical presentation, ranging from varying degrees of cardiac dysfunction to a wide spectrum of extra-cardiac comorbidities, such as liver and kidney damage. These coexisting conditions profoundly influence patient survival and overall prognosis. Therefore, the ability to accurately predict outcomes is paramount for personalized risk stratification, optimizing patient selection for TTVI, and guiding tailored treatment decisions. This predictive capability is particularly critical given the current landscape, where randomized controlled trials for TTVI are still emerging, underscoring the need to identify specific patient subgroups most likely to derive benefit from these interventions.

Clinical evidence suggests that TTVI offers survival benefits and reduces heart failure rehospitalizations when compared to conservative medical management. Notably, patients with mid-range RV function (defined by TAPSE between 13–17 mm) appear to derive the highest treatment effect from TTVI. Conversely, the presence of preexisting RV remodeling (RVR), defined as RV dilation, is associated with increased mortality and rehospitalization rates, despite the observed symptomatic improvement post-TTVR. This highlights the complex interplay between baseline RV state and post-procedural outcomes, emphasizing the need for sophisticated predictive tools [32,33,34].

### 4.2. Biomechanical and Molecular Markers for RVRR Prediction

The quest for accurate outcome prediction in TTVI has led to the exploration of both circulating molecular biomarkers and advanced imaging-derived biomechanical markers.

Among circulating biomarkers, miRNA-486 shows considerable promise as a noninvasive diagnostic marker for RV remodeling, particularly in the context of pulmonary hypertension [35]. Elevated levels of this miRNA are associated with maladaptive RV function, reduced TAPSE/PASP ratios, and increased BNP levels. Its diagnostic performance has been found to be comparable to that of BNP, a widely used biomarker for heart failure, suggesting it could serve as a potential lower-cost alternative. Another significant molecular marker is insulin-like growth factor binding protein 2 (IGFBP-2). Elevated baseline IGFBP-2 levels have been identified as an independent predictor for the non-development of RVRR and persistent TR/RV dilation after transcatheter mitral valve repair [36]. Additionally, IGFBP-2 and circulating miRNA have shown some prognostic value in cardiovascular diseases in general [37,38,39]. Nevertheless, while representing promising novel biomarkers, they certainly have to be confirmed in large registries or trials to confirm their validity. This finding suggests its potential utility in predicting outcomes following tricuspid valve interventions as well.

Advanced imaging modalities provide crucial biomechanical markers for RVRR prediction. 3D-TAPSE derived from CT scans offers incremental value in predicting cardiovascular outcomes, including hospitalization and mortality, after TTVI. Specifically, a posterior iTAPSE (indexed TAPSE) greater than 4.5 mm/m^2^ was associated with significantly lower rates of death and rehospitalization (17.2% vs. 63.6%). Similarly, an iTAPSE volume exceeding 9 mL/m^2^ was linked to a better outcome (16.4% vs. 57.1%). This underscores the critical importance of detailed 3D biomechanical assessment, which often surpasses the predictive capabilities of conventional 2D measures. Furthermore, preserved RV free wall longitudinal strain has been identified as a predictor of RVRR [40,41].

The combination of molecular biomarkers, which reflect the underlying biological processes of maladaptation or recovery, and advanced biomechanical imaging markers, which capture structural and functional changes, offers a more robust and comprehensive approach to predicting RVRR and patient outcomes. This multi-modal strategy moves towards precision medicine, enabling clinicians to select patients more effectively for TTVI and anticipate their response to therapy.

### 4.3. Leveraging Neural Networks and AI for Prognostication

Artificial intelligence (AI) and machine learning (ML) are rapidly transforming the landscape of cardiovascular care, offering significant enhancements in diagnostic accuracy, risk stratification, and outcome prediction [42]. These technologies possess the capacity to analyze vast amounts of patient data with unparalleled accuracy, surpassing traditional analytical methods [43]. In the context of transcatheter tricuspid valve interventions (TTVI), supervised machine learning models are being applied to preprocedural clinical, laboratory, echocardiographic, and hemodynamic data to predict long-term outcomes, including 2-year mortality. A notable example is the survival tree-based model developed by Fortmeier et al., which identified four key parameters for 2-year mortality prediction: mean pulmonary artery pressure (mPAP), N-terminal pro–B-type natriuretic peptide (NT-proBNP) levels, right atrial (RA) area, and estimated glomerular filtration rate (eGFR). This model effectively stratifies patients into distinct risk categories: a low-risk group (mPAP ≤ 28 mmHg and NT-proBNP ≤ 2728 pg/mL) exhibiting an 85.5% 2-year survival rate and a high-risk group (mPAP > 28 mmHg, RA area > 32.5 cm^2^, and eGFR ≤ 51 mL/min) with a significantly worse 52.6% 2-year survival. Other machine learning models, including penalized Cox proportional hazard regression, random survival forest, and extreme gradient boosting, utilize a broader array of clinical and echocardiographic features (e.g., demographics, vitals, comorbidities, laboratory parameters, LV/RV function, TR severity, RV systolic pressure, inferior vena cava size) to predict long-term mortality in TR patients, demonstrating good overall performance. Furthermore, AI also significantly augments imaging analysis [44]; AI-augmented software, such as heart.ai by LARALAB, automates the often cumbersome and time-consuming manual post-processing of CT images, precisely quantifying right heart morphology and 3D-TAPSE measurements throughout the cardiac cycle, thereby enhancing diagnostic precision and enabling a more nuanced biomechanical assessment. These advancements underscore the practical application of AI in refining risk assessment and guiding personalized patient management [32,45].

AI also significantly augments imaging analysis. AI-augmented software, such as heart.ai, automates the often cumbersome and time-consuming manual post-processing of CT images. This software precisely quantifies right heart morphology and 3D-TAPSE measurements throughout the cardiac cycle. This automation enhances diagnostic precision and enables a more nuanced biomechanical assessment, as evidenced by the predictive value of CT-derived 3D-TAPSE parameters [40]. Furthermore, AI can identify tricuspid regurgitation severity from echocardiograms with an accuracy comparable to that of experienced cardiologists and MRI results [46]. The future potential of this technology includes predicting outcomes after heart valve disease treatment, including RVRR. Deep learning models can also accurately predict RVEF from 2D echocardiographic videos, improving prognostication after transcatheter edge-to-edge repair for mitral regurgitation, with potential applicability to tricuspid interventions.

AI’s ability to integrate diverse data types—clinical, laboratory, and multi-modal imaging, including complex biomechanical markers—and identify subtle patterns makes it indispensable for personalized cardiology in TTVI (Table 4). This moves beyond simple risk scores to create dynamic, data-driven prognostic models. Such capabilities allow for more precise patient selection, potentially aiding in real-time procedural guidance and optimizing post-procedural management, ultimately improving patient outcomes. The potential for AI to make TTVI more cost-effective by targeting those most likely to benefit is also being explored.

Despite these advancements, several challenges persist in the integration of AI into clinical practice. The effectiveness of AI models is highly dependent on the quality, robustness, and representativeness of the training data; biases in data or lack of standardization in cardiac imaging can hinder widespread adoption. Many AI models, particularly those based on deep learning, suffer from a “black box” problem, lacking transparency in their decision-making processes, which can impede clinician trust and adoption. (Table 4) The development of explainable AI (XAI) tools, such as probabilistic graphical models, is crucial for addressing this limitation and building confidence in AI-driven risk stratification. Furthermore, rigorous prospective studies in diverse populations and clear regulatory guidelines are essential for clinical validation and widespread implementation. Practical barriers also include the initial costs of implementation and the challenges of integrating AI solutions into existing electronic health records and clinical workflows, although phased rollouts and embedded decision support tools may facilitate this process. Ethical considerations, including data privacy, algorithmic bias, and equitable access to these advanced technologies, must also be carefully addressed [47,48].

## 5. Conclusions and Future Perspectives

The right ventricle’s response to tricuspid regurgitation involves a complex, multi-scale remodeling process driven by intricate molecular and biomechanical maladaptations. Key molecular drivers include an early failure of antioxidant defenses and increased mitochondrial ROS production, a metabolic shift towards glycolysis (Warburg effect) with impaired fatty acid oxidation, and dysregulation of various microRNAs. Biomechanically, the RV undergoes a transition from adaptive concentric hypertrophy to maladaptive eccentric dilation, accompanied by increased tissue stiffness, fibrosis, and detrimental myofiber reorientation. These changes collectively impair RV function and uncouple it from the pulmonary circulation.

Transcatheter tricuspid valve interventions have emerged as a transformative therapeutic modality, effectively reducing TR severity and initiating a beneficial, often biphasic, right ventricular reverse remodeling. This remodeling is characterized by an early reduction in RV volume overload, followed by later structural changes, alongside improvements in RV function, myocardial strain, and RV–pulmonary arterial coupling. This dynamic process underscores the critical link between mechanical unloading and subsequent biological and structural recovery.

The inherent heterogeneity of patients with severe TR and the high-risk nature of their condition necessitate accurate outcome prediction for personalized treatment strategies. Emerging molecular biomarkers, such as circulating miRNA-486 and IGFBP-2, alongside advanced imaging-derived biomechanical markers like CT-based 3D-TAPSE and RV longitudinal strain, are proving valuable in identifying patients most likely to experience RVRR and favorable clinical outcomes (Figure 1).

Artificial intelligence and machine learning are poised to revolutionize outcome prediction in this domain. By integrating diverse clinical, laboratory, and advanced imaging data, AI models can overcome the limitations of traditional assessment methods, enabling unprecedented precision in personalized risk stratification and optimizing treatment strategies for TTVI. AI-augmented analysis of imaging modalities, for instance, automates complex measurements and provides nuanced biomechanical insights that are critical for prognostication. This represents a significant step towards precision cardiology, allowing clinicians to select patients more effectively, anticipate their response to therapy, and potentially enhance the cost-effectiveness of these interventions.

Future research directions must prioritize the standardization of cardiac imaging data acquisition and reporting, particularly for the RV, to facilitate the development and robust validation of AI models across diverse patient populations. A deeper mechanistic understanding of the molecular and cellular pathways driving RVRR post-TTVI is also essential, moving beyond observational correlations to elucidate causal relationships. The development of explainable AI (XAI) tools is crucial to foster clinician trust and facilitate widespread adoption. Large-scale, prospective, and ideally randomized (SHAM)-controlled trials are indispensable for validating the clinical outcome improvement and cost-effectiveness of TTVI in general. Ongoing trials such as TRICI-HF, TRICEND II, LEAFLET II Trial will give us more insights into the effectiveness and outcome of patients undergoing transcatheter tricuspid valve interventions. Furthermore, the continued development of advanced robotic RV models offers a promising platform for in vitro testing of interventions and a more profound understanding of biomechanical improvements. Finally, sustained long-term follow-up studies are necessary to assess the durability of RVRR and its enduring impact on patient survival and quality of life.

## Figures and Tables

**Table 1:** Key Molecular Mechanisms in RV Maladaptive Remodeling.

Category	Molecular Factor	Role in Maladaptive Remodeling	Potential for RVRR
Oxidative Stress and Metabolism	Reactive Oxygen Species (ROS)	Increased production; early failure of antioxidant defenses (SOD, GPX) in RV vs. LV; greater mitochondrial ROS generation. Leads to damage and apoptosis.	Reduction in oxidative stress (e.g., with antioxidants like EUK-134) improves RV systolic function.
	PGC1α	Decreased expression, leading to impaired fatty acid oxidation, reduced mitochondrial mass/number, decreased oxidative capacity, increased ROS, mitochondrial DNA damage.	Upregulation could restore metabolic function.
	HIF-1α	Activation associated with complex II-mediated ROS production in RVH; impaired angiogenic response (decreased VEGF, unchanged capillarity).	Modulation could improve angiogenesis and reduce ROS.
	Pyruvate Dehydrogenase Kinase (PDK)	Increased expression mediates shift to aerobic glycolysis (Warburg effect), reducing ATP efficiency and RV contractility.	Pharmacologic inhibition improves RV contractility.
	Fatty Acid Metabolism (Dysfunctional)	Decreased fatty acid oxidation, increased lipid accumulation, production of toxic intermediates (ceramide, palmitate), lipotoxic cardiomyopathy.	Restoration of fatty acid oxidation.
Angiogenesis and Epigenetics	MicroRNAs (miR-143/145, miR-34, miR-379, miR-503, miR-126, miR-486)	Dysregulation in RV failure (vascular tone, apoptosis, endothelial proliferation, VEGF pathway inhibition).	Targeted delivery (e.g., miR-126) shows increased RV vascularity/function. Circulating miR-486 is a diagnostic biomarker for maladaptive RV remodeling.
	IGFBP-2 (Insulin-Like Growth Factor Binding Protein 2)	Elevated levels at baseline predict non-development of RVRR and persistent TR/RV dilation after M-TEER.	Lower levels associated with RVRR.
Signaling Pathways	PI3K/Akt/mTOR pathway	Inactivated in pathological hypertrophy (pressure overload).	Activated in physiological hypertrophy (exercise-induced); enhances ventricular hypertrophy and function, suppresses apoptosis; gene therapy with constitutively active PI3K can improve function.

**Table 2:** Advanced Imaging Modalities for RV Function and RVRR Assessment.

Modality	Strengths	Parameters Measured	Utility for RVRR	Limitations
Echocardiography (2D and 3D)	First-line, widely available, real-time, non-invasive. 2D strain (speckle tracking) is angle-independent, sensitive for early dysfunction, good reproducibility. 3D echo provides comprehensive anatomical and volumetric assessment.	TAPSE, RVFAC, RVMPI, RV-Sa (conventional). RV peak systolic strain (RVPSS), RV global longitudinal strain (RV GLS). RV volumes (RVEDV3D, RVESV3D), RVEF3D (3D echo).	Early detection of myocardial deformation impairment. Quantifies RV volume unloading and structural remodeling. Measures improvement in RV FW-GLS and RV-PA coupling (TAPSE/PASP).	2D methods limited by suboptimal acoustic windows, geometric assumptions, and inability to capture complex 3D anatomy. 2D-TAPSE alone may fail to predict outcomes.
Cardiac Magnetic Resonance Imaging (CMR)	Gold standard for volumetric quantification of RV. Excellent endocardial definition, no ionizing radiation. Quantitative TR measurement via phase-contrast imaging. Can assess regional RV performance with tissue tagging.	RV volumes (end-diastolic, end-systolic), RVEF, RV mass. TR regurgitant volume, TR fraction. Regional shortening.	Precise quantification of RV volume reduction and changes in EF. Baseline RVESV (CMR-derived) is a strong predictor of RVRR.	Impractical for some patients (inability to stay supine, implanted devices). Less available than echo.
Cardiac Computed Tomography (CT)	Detailed anatomy of TV and RV. Full-cycle CT captures complex RV anatomy and annular plane dynamics. AI-augmented software automates post-processing and quantification.	RV volumes (RVEDV, RVESV), RVEF. CT-based 3D-TAPSE (anterior, posterior, septal, lateral), iTAPSE, iTAPSE volume.	Substantial reduction in RV-EDV after TTVR (35%). Posterior iTAPSE and iTAPSE volume are independent predictors of cardiovascular outcomes after TTVI.	Ionizing radiation. Contrast use.

**Table 3:** Biomechanical Changes and Markers of RVRR Post-TTVI.

Biomechanical Change/Marker	Observed Change	Impact on RV
Tricuspid Regurgitation (TR) Severity Reduction	Immediate and significant reduction in TR grade (e.g., to ≤2+ in 83% of patients at 6 months, 41% reduction in vena contracta, 50% in TR volume, 54% in EROA). Optimal procedural results (residual TR ≤ 1+) associated with more pronounced RVRR.	Reduces RV volume overload and wall stress, improving cardiac efficiency.
RV Volume Reduction (Reverse Remodeling)	Biphasic pattern: early RV volume unloading (RVEDV reduction, e.g., −9.7% at discharge, −35% after TTVR) and later structural remodeling (RVESV reduction, e.g., −5.4% at 6 months). Average RV and TV dimensions decrease significantly.	Improved RV geometry, reduced wall stress, and enhanced cardiac output. Associated with improved survival.
RV Ejection Fraction (RVEF)	May initially decline post-TTVI but gradually increases over time, returning to baseline values by 2 years. Effective RVEF improves immediately post-procedure.	Reflects improved pump function and overall RV efficiency.
RV Global Longitudinal Strain (RV GLS)	Initial decline followed by late recovery to pre-procedural baseline values. Improvement in RV FW-GLS is a definition of RVRR (>10% improvement).	Direct assessment of myocardial deformation, indicating improved contractility and less impairment.
RV-Pulmonary Artery (RV-PA) Coupling (TAPSE/PASP ratio)	Significant improvement (e.g., from 0.36 to 0.42). Improvement in RV-PA coupling is a definition of RVRR (>10% improvement).	Reflects improved RV efficiency in handling afterload, crucial for prognosis.
Biventricular Interaction	Reduction in RV volume overload improves biventricular interaction, alleviating leftward bowing of the septum and improving early LV filling. Increased LV forward stroke volume (e.g., 30% increase).	Enhanced overall cardiac output and systemic perfusion.

**Table 4:** AI/Machine Learning Models for Predicting TTVI Outcomes.

Model Type	Input Data Types	Key Input Parameters (Examples)	Risk Stratification (Example Cut-Offs)	Model Output	Utility/ Significance
Survival Tree-Based Model	Preprocedural clinical, laboratory, echocardiographic, and hemodynamic data.	Mean pulmonary artery pressure (mPAP), NT-proBNP levels, Right Atrial (RA) area, Estimated Glomerular Filtration Rate (eGFR).	Low-Risk: mPAP ≤ 28 mmHg AND NT-proBNP ≤ 2728 pg/mL (2-year survival: 85.5%) High-Risk: mPAP > 28 mmHg AND RA area > 32.5 cm^2^ AND eGFR ≤ 51 mL/min (2-year survival: 52.6%)	2-year survival rate.	Effectively stratifies patients into distinct risk categories, comparable to TRI-Score and outperforms EuroScore II in identifying high-risk patients. Informs patient selection and personalized treatment.
Penalized Cox Proportional Hazard Regression, Random Survival Forest (RSF), Extreme Gradient Boosting	27 clinical and echocardiographic features from echocardiography reports and electronic medical records.	Heart rate, right ventricular systolic pressure (RVSP), blood pressure, diuretic use, age, BMI, chronic kidney disease, prior cardiac surgery, signs of congestion/hypoperfusion (AST, creatinine, hyponatremia), LV ejection fraction, LV end-diastolic dimension.	Identifies top contributing features for mortality prediction.	1-year and 3-year mortality prediction (C-index 0.74–0.75 for 1-year).	Good overall performance in predicting long-term mortality in TR patients. Conditional RSF often ranks highest.
Deep Learning for RVEF Prediction from 2D Echo	2D apical 4-chamber view echocardiographic videos.	Video frames processed by convolutional networks.	Reduced RVEF < 45% (significantly worse 1-year survival: 80.3% vs. 92.1%).	Predicted RVEF.	Refines prognostication, superior to conventional 2D TAPSE in predicting 1-year mortality. Can screen for patients needing intensified follow-up.
AI-augmented CT analysis for 3D-TAPSE	Full cardiac cycle CT images (axial thin slices).	3D tricuspid annulus dynamics (anterior, posterior, septal, lateral TAPSE measurements, iTAPSE volume).	Posterior iTAPSE > 4.5 mm/m^2^ (1-year combined endpoint: 17.2% vs. 63.6%). iTAPSE volume > 9 mL/m^2^ (1-year combined endpoint: 16.4% vs. 57.1%).	CT-based 3D-TAPSE values, prediction of hospitalization and mortality.	Automates complex measurements, provides incremental predictive value over 2D-TAPSE, refines risk stratification.

## Abbreviations

AI	Artificial intelligence
CMR	Cardiac magnetic resonance imaging
CT	Computed tomography
eGFR	Estimated glomerular filtration rate
IGFBP-2	Insulin-like growth factor binding protein 2
LV	Left ventricular/left ventricle
miRNAs	MicroRNAs
mPAP	Mean pulmonary arterial pressure
NT-proBNP	N-terminal pro–B-type natriuretic peptide
PASP	Pulmonary artery systolic pressure
PDK	Pyruvate dehydrogenase kinase
RA	Right atrial/right atrium
ROS	Reactive oxygen species
RV	Right ventricular/right ventricle
RVEDV	Right ventricular end-diastolic volume
RVEF	Right ventricular ejection fraction
RV GLS	Right ventricular global longitudinal strain
RV-PA	Right ventricular-pulmonary arterial
RVESV	Right ventricular end-systolic volume
RVRR	Right ventricular reverse remodeling
T-TEER	Transcatheter tricuspid valve edge-to-edge repair
TAPSE	Tricuspid annular plane systolic excursion
TR	Tricuspid regurgitation
TTVI	Transcatheter tricuspid valve interventions
TTVR	Transcatheter tricuspid valve replacement
